# Chromatic adaptation from achromatic stimuli with implied color

**DOI:** 10.3758/s13414-019-01716-5

**Published:** 2019-06-14

**Authors:** R. J. Lee, G. Mather

**Affiliations:** School of Psychology, University of Lincoln, Lincoln, UK

**Keywords:** Color and light: color, Color and light: constancy, Adaptation and aftereffects

## Abstract

Previous research has shown that the typical or memory color of an object is perceived in images of that object, even when the image is achromatic. We performed an experiment to investigate whether the implied color in greyscale images could influence the perceived color of subsequent, simple stimuli. We used a standard top-up adaptation technique along with a roving-pedestal, two-alternative spatial forced-choice method for measuring perceptual bias without contamination from any response or decision biases. Adaptors were achromatic images of natural objects that are normally seen with diagnostic color. We found that, in some circumstances, greyscale adapting images had a biasing effect, shifting the achromatic point toward the implied color, in comparison with phase-scrambled images. We interpret this effect as evidence of adaptation in chromatic signaling mechanisms that receive top-down input from knowledge of object color. This implied color adaptation effect was particularly strong from images of bananas, which are popular stimuli in memory color experiments. We also consider the effect in a color constancy context, in which the implied color is used by the visual system to estimate an illuminant, but find our results inconsistent with this explanation.

**Electronic supplementary material:**

The online version of this article (10.3758/s13414-019-01716-5) contains supplementary material, which is available to authorized users.

## Introduction

The mechanisms in the peripheral visual pathway that support human color vision are reasonably well understood: signals from three classes of cone photoreceptor (long-, medium-, and short-wavelength sensitive, or L, M, and S), tuned to different regions of the visible spectrum are combined by specific populations of ganglion cells in the retina in an opponent fashion. These ganglion cells project to the lateral geniculate nucleus (LGN) and from there signals are sent to the cortex. However, it has been suggested that what we see is not only determined by the optic array (the pattern of light reaching our eyes) but is also partly determined by knowledge or prior experience of the world and an estimate of the relative likelihood of possible stimuli. The debate on the bottom-up vs. the top-down nature of vision has been ongoing through much of modern vision science and continues today (Firestone & Scholl, 2016). Relatively recently, more evidence had been provided for top-down inputs to color perception, and we discuss some of this below. In the experiment we describe here, we attempt to determine whether the typical bias from expected colors of objects, known to affect color vision when that color is not present, lasts beyond the presentation of that stimulus, in a way analogous to traditional chromatic adaptation in the peripheral mechanisms.

### Memory color affects color perception

For some time, it has been suggested that the remembered typical color of an object—“memory color” (as opposed to “color memory”, simply remembering colors, Bartleson 1960; Hering 1964)—can affect the perceived color of that object. One of the earliest people to make this suggestion was Hering (1964, p. 8), who went as far as to suggest that the memory color seen in a “fleeting glance” could replace the perception of another color, depending on attention. Several studies have shown that the typical or diagnostic color (Tanaka & Presnell, 1999; Biederman & Ju, 1988) of an object can have an effect on the perception of that object’s color. These experiments use stimulus images featuring natural objects (often fruit and vegetables, Bannert & Bartels 2013; Hansen, Olkkonen, Walter, & Gegenfurtner, 2006; Olkkonen, Hansen, & Gegenfurtner, 2008) that have a typical color, objects that are familiar to the particular observer (Bloj, Weiß, & Gegenfurtner, 2016), and/or objects with popular branding or symbolism (e.g., road signs, Witzel, Valkova, Hansen, & Gegenfurtner, 2011). The effects can go beyond the perceived color of the object, for example it has been shown that scenes are better recognized when they contain objects in their diagnostic colors (Goffaux, Jacques, Mourax, Oliva, Schyns, & Rossion, 2005). The perceived colors of after-images, created by viewing color-inverted images of recognizable objects, have also been shown to be affected by the expected color of the object, and this can be modulated by an estimate of the precision of the signal (which is low for after-images, as they are unstable, Lupyan^2015^).

In experiments in which observers were asked to adjust an image of an object with diagnostic color to be achromatic, they usually selected colors that were opposite (on the opposite side of the achromatic point in color space) from that color (Hansen et al., 2006; Olkkonen et al., 2008; Witzel et al., 2011; Lupyan, 2015). This is consistent with having to ‘over-compensate’ to negate the chromatic perception caused by the familiar color of the object. This effect was reduced when the stimuli were made less realistic by having their texture reduced or made into outline shapes only (Olkkonen et al., 2008). In addition, the color of the objects used in these experiments is remembered with more saturation and lightness than typical examples (Bartleson, 1960; Delk & Fillenbaum, 1965) or the specific items known to the observer (Bloj et al., 2016).

When greyscale images of color-diagnostic images are shown to observers, it has been shown that the memory color can be predicted from neural activity in the primary visual cortex (V1, Bannert & Bartels 2013) and the authors concluded that this region is receiving signals about the prior knowledge of object colors, possibly as feedback from area V4.

### Color constancy

We see objects because light from an illumination source reflects from their surface and into our eyes. The spectral composition of this light is determined by both the illuminant and the surface reflectance properties, but in general we see surfaces as being the same color in different illumination conditions. This is one, perhaps too restricted, definition of color constancy (Smithson, 2005; D’Zmura & Lennie, 1986; Foster, 2011; Hurlbert, 1998). The problem of recovering the surface properties is a difficult one for the visual system, and there are many suggestions of how the illuminant might be estimated in order to be discounted from the proximal stimulus, using chromatic information distributed in space or time in the stimulus. Another way that this might be done is by comparing the chromatic signal to the expected color determined by the diagnostic color of an object, and assuming the difference is caused by the chromaticity of the illuminant. This was also part of Hering’s (1964, p. 17) observations on memory colors. A bias of the signal towards the expected object color could then be applied to the rest of the scene to achieve color constancy. Memory color has been experimentally investigated in a color constancy context, and any effect of familiar objects is small (Granzier & Gegenfurtner, 2012; Kanematsu & Brainard, 2014).

### Chromatic adaptation

Much of what we know about the peripheral mechanisms of color vision comes from studying adaptation or habituation. Continued stimulation of a particular mechanism reduces the activity of that mechanism relative to others. This can be done selectively and the effects measured in psychophysical experiments (see for reviews Clifford et al., 2007). It can readily be shown that adaptation to a colored stimulus for only a few seconds produces negative after images that also last seconds. This kind of adaptation, which we-refer to as “normalization”, has the effect of making the adapting stimulus appear closer to neutral and occurs in systems where the stimulus dimension is encoded by broadband mechanisms (such as the spectral sensitivities of the cones), rather than a larger number of narrowly tuned mechanisms (see Webster 2011, for discussion) where the effect can sometimes be opposite.

In color vision, adaptation can be shown to both the time-averaged magnitude of a stimulus and to the amount of variation (contrast adaptation, see Webster 1996, for review), and has been used as a tool to study the nature of processing in the system. For example, Krauskopf, Williams, and Heeley (1982) adapted observers to chromatic modulations in different directions in color space and demonstrated that thresholds for detecting subsequent colors could be elevated selectively in only two directions—the cardinal axes of color space, which are determined by the responses of the opponent mechanisms. The comparison of cone signals by different classes of retinal ganglion cells (RGCs), which project to the lateral geniculate nucleus (LGN), results in the perceptually opposite colors. The L-M mechanism roughly encodes redness vs. greenness, and the S-(L+M) mechanism roughly encodes blueness vs. yellowness, although these colors are not the same as the unique hues. The color visual system has a perceptual “norm”, when signals might be considered in balance and stimuli appear achromatic (Webster & Leonard, 2008), although this can vary between individuals. In the experiment we describe here, we are particularly concerned with the first kind of adaptation, to the steady or time-averaged stimulus. Viewing a predominantly yellow stimulus for a few seconds, for example, produces a blue after-image when subsequently viewing an objectively neutral field. The effect is most easily demonstrated with a discrete stimulus that creates an after-image, but a chromatically biased scene will have the effect of adapting the whole visual field, producing a perceptual color bias. This kind of normalization has been shown to result from gain-control-like processes in various stages of the chromatic pathway, including in the cones themselves (Valeton & van Norren, 1983) and the RGCs (Zaidi, Ennis, Cao, & Lee, 2012), although other observed effects suggest that a component of adaptation is cortical (e.g., Shimojo, Kamitani, & Nishida, 2001; Zeki, Cheadle, Pepper, & Mylonas, 2017). Further experiments, including reanalysis of the original data from (Krauskopf et al., 1982) by Krauskopf, Williams, Mandler, and Brown (1986), suggest that there are additional mechanisms, possibly later in the pathway than the opponent ones that are tuned to different hues (Eskew, 2009), but little is known about how adaptation in these “higher-order mechanisms” would affect perception.

Adaptation is traditionally thought of as being driven by bottom-up input from sensation. However, in this experiment, we are more interested in the potential adapting effects from top-down input, originating from memory color. We would not like to speculate about whether this occurs at any of the stages of the system that we know of, and it does seem unlikely that it occurs in any of the peripheral, opponent mechanisms. Nevertheless, the LGN does receive input from V1 (Fitzpatrick, Usrey, Schofield, & Einstein, 1994), so it is not impossible that top-down driven adaptation does take place here.

Adaptation takes place in many perceptual modalities, not limited to color or even vision. Mechanisms sensitive to stimulus motion can be adapted with a stimulus with continuous motion in one direction, so that subsequent stationary stimuli appear to drift in the opposite direction to the adapter (Mather, Verstraten, & Anstis, 1998). Particularly relevant to our current work, viewing static photographs of moving subjects (e.g., running figures) is also claimed to produce motion adaptation, affecting perceived direction of subsequent stimuli composed of drifting dots (Winawer, Huk, & Boroditsky, 2008). These stimuli with implied motion have an effect analogous to the effect we aimed to produce in our experiment, but it has been said that the measured effect is actually a result of a shift in the observers’ decision criterion, not a perceptual bias (Morgan, Melmoth, & Solomon, 2013; Mather & Sharman 2015). We use a methodology designed to avoid this problem and measure a true perceptual bias, as will be described later.

### Rationale

We might expect that viewing images of objects that have a typical, diagnostic color will affect the perception of not just the chromaticities in those images, but also the chromaticities in subsequent stimuli, even when those images of objects are made achromatic. We suggest two subtly different reasons why this might be the case, that each cause chromatic biases in different directions: ‘normalization’ and ‘illuminant compensation’. Firstly, knowledge of color might provide input to color vision mechanisms at some level—not necessarily the peripheral mechanisms mentioned above—adjusting their gain to cause a chromatic bias in perception that lasts beyond the adaptors. A greyscale image of a typically yellow object might provide excitation in a mechanism that responds to yellow, in a similar (but likely much reduced) way to a naturally colored image. Adaptation will occur, the output of the mechanism will be reduced, and subsequent stimuli will then appear biased away from yellow—towards blue according to opponent models of color perception—neutral stimuli would appear more blue or yellow stimuli would appear more neutral. This could also be considered as a shift in the neutral point towards yellow. Alternatively, if the visual system uses memory color to estimate illuminant chromaticity in order to discount it, then viewing the greyscale image of a typically yellow object would suggest an extremely blue-biased illuminant, resulting in an achromatic reflection. Subsequent stimuli might still be perceived as if under this blue illuminant, if presented within a short time afterwards, as it takes the visual system some time to adjust to a different illuminant (Lee, Dawson, & Smithson, 2012). The perceptual effect of the resulting compensation would be opposite to the effect of the normalization just described. Subsequent neutral chromatic stimuli would appear yellow, just as the adapting stimulus was, so the overall biasing effect is yellow (or a shift in the neutral point towards blue). In this experiment, we aimed to directly test whether achromatic images of typically colored objects, images with implied color, can cause normalization-like or illuminant-compensation-like perceptual chromatic biases in subsequent, simple, stimuli. The chromatic direction of any such bias might indicate which of the above routes is the cause. However, we do not anticipate that the stimuli we use will be particularly effective in causing an illuminant bias, for reasons we discuss later, nor is illuminant discounting the only way that color constancy might be achieved. We used a standard top-up adaptation experiment paradigm, with methodology designed to remove the effect of response bias or any strategy based on the semantic content of the images.

## Method

### Observers

Four observers participated in this experiment. One was male, the others female. All had normal color vision (as verified with Ishihara’s Test for Color Deficiency), and normal or corrected-to-normal acuity. One observer was one of the authors, and has knowledge of color theory and color space, and another observer was experienced with psychophysics experiments but not color, specifically. The final two observers were less experienced in psychophysical observations. Each observer contributed between 5 and 16 hours of time to data collection. The study received clearance from University of Lincoln school of Psychology Research Ethic Committee.

### Stimuli

**Fig. 1:**
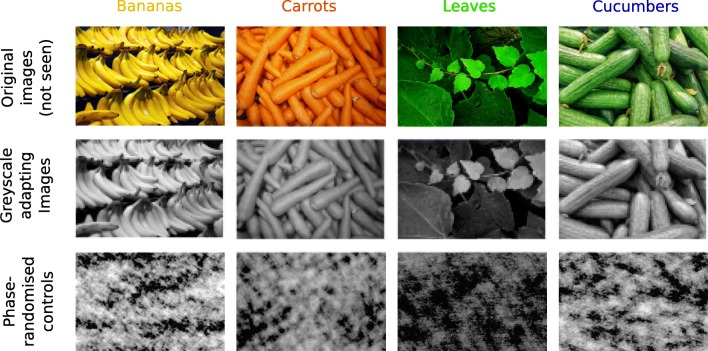
Examples of the adapting images used in the experiment. Each column shows an image from one of the four sets (bananas, carrots, leaves, cucumbers), and each row shows the different modifications of each image. The top row shows the original images in full color, which were not used in the experiment. The middle row shows the original images in greyscale, as used in one condition of the experiment. The bottom row shows the phase-scrambled versions of the images in the middle row, as used in the control conditions

Adapting images were images of natural objects. The objects were chosen because they are predominantly a particular color, yet can still be identified when they are greyscale. The objects we selected were: bananas, carrots, leaves, and cucumbers (Fig. 1). In the first round of data collection, we used only the first three objects. The cucumber images were used later, in an attempt to understand unexpected effects from the leaves. The original images to be used as adaptors were found from Internet searches, and chosen because they met the following criteria: The images contained many examples of the object, such that the whole image was filled with examples; there were no other objects in the scene; the images were of sufficiently high pixel resolution that they did not appear pixelated on the experiment monitor. We made these decisions because we wanted images that did not contain other objects or surfaces that might provide a reference against which the color of the adapting objects may be judged or the illuminant estimated. Some images found that met these requirements were very large, and were split to create more than one stimulus image. Images were cropped as necessary to fit the 4:3 aspect ratio of the experimental monitor. Finally, the images were converted from RGB color to greyscale (MATLAB’s rgb2gray function), and scaled in luminance so that the mean of all adapting images was the same, and the same luminance as the probe stimuli (see below).

The adapting images for the control conditions were made by randomizing the phases of the Fourier components of the original images. The control images therefore contained the same Fourier amplitude components, and approximately the same luminance distributions, as the original images.

**Fig. 2:**
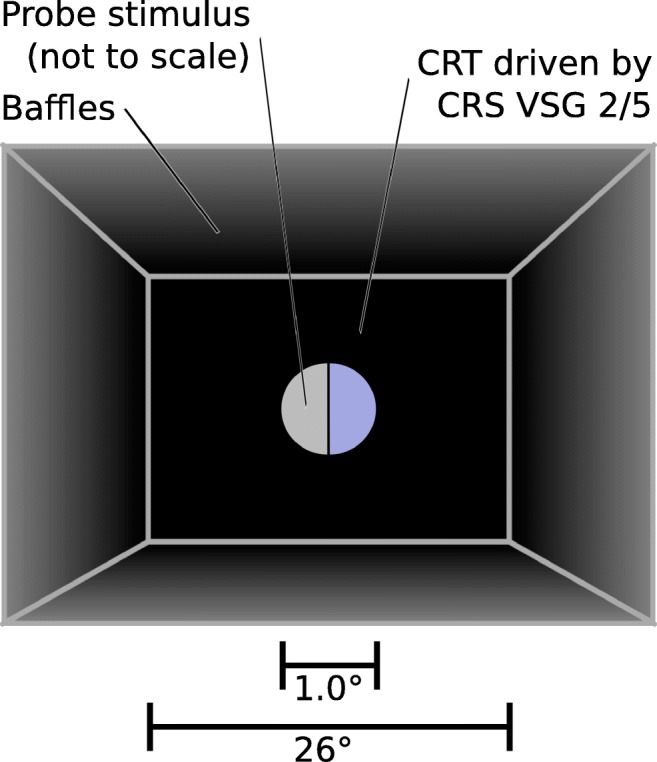
The configuration for the apparatus in the experiment, from the point of view of the observer. The monitor is surrounded by black card baffles to remove any view of the rest of the room. The colour probe stimulus is shown, not to scale

Probe stimuli were comprised of a disc, approximately 1° visual angle in diameter, divided into semicircles by a narrow vertical black line (see Fig. 2). The two segments were assigned chromaticities from either a ‘pedestal’ or a corresponding ‘pedestal plus test’ set.

All colors for the probe stimuli were defined in CIE *L*^∗^*a*^∗^*b*^∗^ space and converted to appropriate RGB values. CIE *L*^∗^*a*^∗^*b*^∗^ was used, as it is intended to be more perceptually uniform—the Euclidean distance between two points corresponds to a perceptual difference that is approximately the same in any part of the space—than any of the spaces based on the physiological mechanisms. Conversion to RGB was done by transforming CIE *L*^∗^*a*^∗^*b*^∗^ values to CIE (1931) XYZ values, and calculating the corresponding RGB values using the spectral measurements of the monitor. The pedestal set had three colors, one was grey (*L*^∗^*a*^∗^*b*^∗^ = (100,0,0)), and the others were slightly red and green in half of the sessions (*L*^∗^*a*^∗^*b*^∗^ = (100,1,0) and (100,− 1,0), respectively) and slightly yellow and blue (*L*^∗^*a*^∗^*b*^∗^ = (100,0,1) and (100,0,− 1), respectively) in the other half of the sessions. There was a separate test set for each pedestal, containing eight offsets from the pedestal, centered on zero offset and equally spaced over 5.0 units in *L*^∗^*a*^∗^*b*^∗^ space in the color direction being used in the session. With this range of colors used, the appearances of the pedestal and test plus pedestal colors were very similar and the task was difficult. One observer in particular was unable to make the perceptual decision with any degree of certainty, and so the range of the chromaticities was doubled for this observer.

The luminance of the grey pedestal, relative to which all other colors were specified, was 26.6*cdm*^− 2^. All other pedestal and test chromaticities had the same luminance, as L* values were the same for all these stimuli.

### Apparatus

All stimuli were presented on a Sony Trinitron G400 CRT driven by a CRS (Cambridge Research Systems, Rochester, Kent, UK) VSG 2/5 providing 14-bit per channel chromatic resolution. The display was gamma-corrected using a CRS ColorCAL, and spectral measurements were made with a JETI Specbos 1211 (JETI Technische Instrumente GmbH, Jena, Germany). Observers viewed the display from a distance of 60 cm while resting on a chin-rest, so the whole display subtended 34 × 26 degrees of visual angle. Observers gave their responses with a button box. Black baffles surrounded the monitor and the observer to prevent stray light from the monitor illuminating other objects in the room, which was otherwise dark (see Fig. 2).

### Procedure

**Fig. 3:**
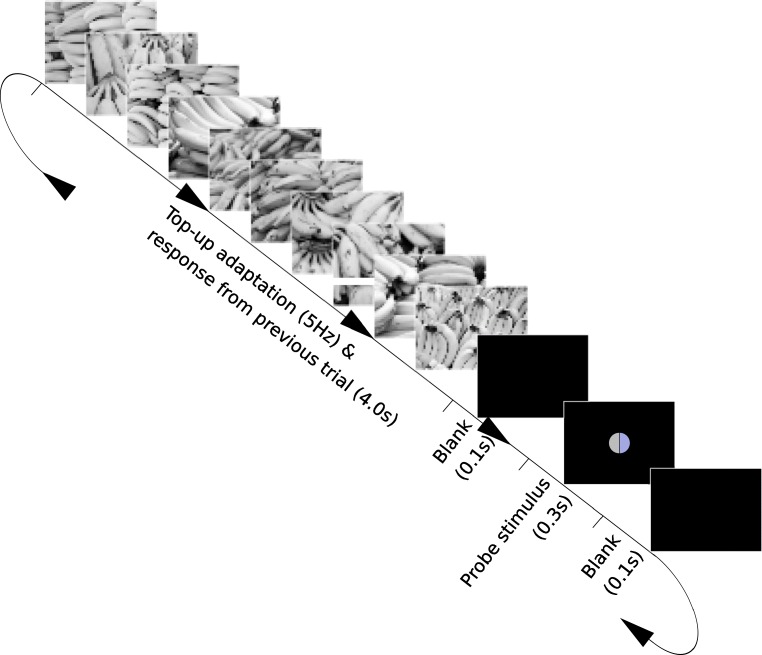
The sequence of stimuli in one trial of the experiment. Greyscale images, either from one of the sets of images of typically colored images, or from the set of phase-scrambled controls, were shown in a random order, 5 per second for 4 seconds. A 0.1-s black frame followed, and this was followed by the probe stimulus (see description in text) for 0.3 s

Each experimental session began with an initial adaptation period of 120s. This consisted of adapting images presented at the rate of 5 per second, in a randomized order. The sequence of trials then began immediately. Each trial consisted of the presentation of adapting images for 4 s (again at 5 Hz in a randomized order). This was followed by a 100-ms black interval, then the probe stimulus for 300 ms, and a second 100-ms black interval. The period of adaptation images for the next trial began immediately (Fig. 3), and the observer had the first 2 s of this period to give their response. The presentation rate meant that each image was displayed for 200 ms, more than long enough for object identification with color photographs and line drawings (Biederman & Ju, 1988), but not long enough to result in after images. The sequence of each trial meant that there was no period where the routine paused to wait for a response, so any adapting effect was maintained as much as possible, but there was enough time between each probe stimulus to make the task possible for the observer.

The observer’s task was to decide which of the two halves of the probe stimulus was least saturated or “most like grey” and respond by pressing the corresponding one of two buttons.

The procedure to measure chromatic bias was analogous to that used by Morgan (2014) and Morgan, Grant, Melmoth, and Solomon (2015) to measure orientation perception bias and by Mather and Sharman (2015) and Morgan, Schreiber, and Solomon (2016) to measure speed perception bias. One-half of the probe was assigned to be the ‘pedestal’ and one was the ‘pedestal plus test’. This assignment was randomly determined for each trial, and the observer was not aware of which was which. In each session, there were three pedestal values used, and eight test values per pedestal. Each combination was repeated ten times per session, giving 240 trials per session. Separate sessions were used for each combination of adapting image set, adapting or control condition (see below) and chromatic direction (*a*^∗^ and *b*^∗^, roughly red-green and yellow-blue). Each session was repeated 1–3 times, giving between 10 and 30 repeats of each unique trial and 3840 to 11,520 total trial per observer. Each session lasted approximately 20 min. Sessions were run in a paired fashion so that a session of the control condition was run with only a short break preceding or following the corresponding adapting images session.

## Results

**Fig. 4:**
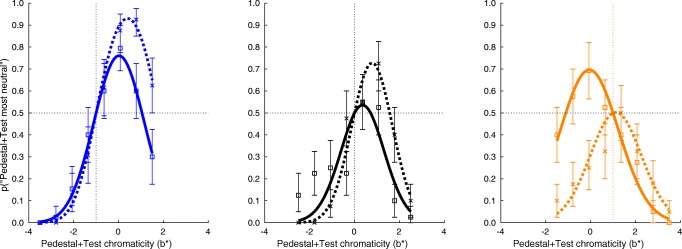
Example response proportions and fitted psychometric functions from one observer, for the *b*^∗^ (yellow-blue) chromatic direction and banana stimuli. Separate panels show data from trials with different pedestal chromaticities. Left panel: blue (*b*^∗^ = − 1). Center panel: neutral (*b*^∗^ = 0). Right panel: yellow (*b*^∗^ = 1). The vertical axes are the proportions of times that the observer responded that the pedestal plus test stimulus (as opposed to the pedestal) was more neutral, for each of the pedestal plus test chromaticities on the horizontal axis. Data for the control condition (phase-scrambled images) are shown with square symbols and fitted with solid curves. Data from the image condition are shown with x symbols and fitted with dashed curves. Vertical dotted lines indicate the chromaticities of the pedestals, and the horizontal dotted line indicates the proportion at which the observer is equally likely to choose the pedestal plus test or pedestal

For each observer, image category (bananas, carrots, leaves, cucumbers), experimental condition (images or phase scrambled control), color direction and pedestal, we calculated the proportion of times the ‘pedestal plus test’ color was chosen as least saturated. These proportions, as functions of the pedestal (*p*) plus test (*t*) chromaticity, were fitted by a modified version of the function used by (Morgan et al., 2016):

$$ \text{Pr}(\text{``}p+t^{\prime\prime}) = \frac{1}{2}\left( 1+\operatorname{erf}\left[\frac{2\mu-p-t}{2\sigma}\right]\operatorname{erf}\left[\frac{t-p}{2\sigma}\right]\right) $$

separately for each of the pedestals and for each of the two *a*^∗^ and *b*^∗^ chromatic axes. Figure 4 shows a representative example of the three functions fitted to data for one observer, image set condition, and color direction. The complete set of plots, including r^2^ values corresponding to the fits, can be found in the Supplementary Material. Some features of these plots are important: if the test were zero (i.e. pedestal and pedestal plus test were the same, which did not actually occur in our experiment) we would expect observers to indicate that the pedestal plus test was least saturated as often as they indicate the pedestal. The fitted curves are constrained to pass through the 0.5 proportion at the chromaticity of the corresponding pedestals (indicated by the vertical dashed lines) and they appear to fit the data well. This anchor need not be the peak of the curve, although it is expected that the peak will be around this point in the case of the control condition with neutral (*a*^∗^ = 0 and *b*^∗^ = 0) pedestals.

**Fig. 5:**
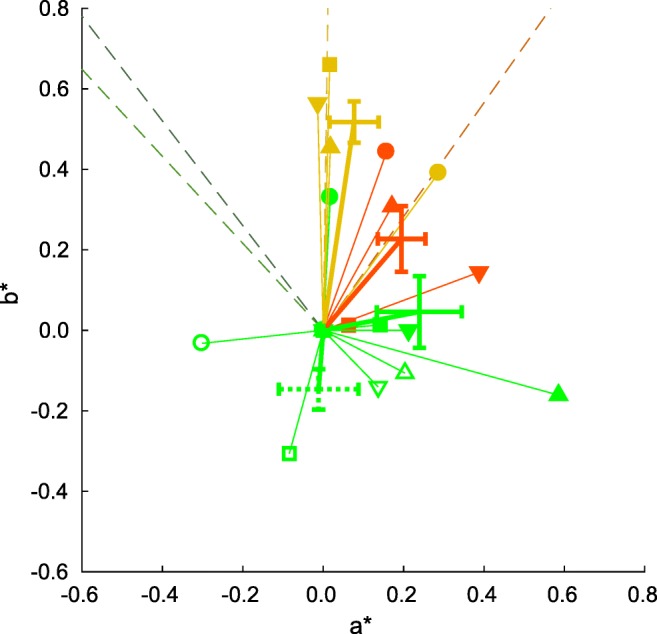
Chromatic biases for all observers in the constant-lightness plane of CIE *L*^∗^*a*^∗^*b*^∗^ space. Each colored symbol, connected to the origin by a thin colored line, shows the chromatic vector representing the perceptual bias resulting from viewing the adapting images. These shifts are calculated from the differences between the mean (*μ*) parameters of the function fitted to response proportions (see Fig. 4). Biases are shown for each observer separately, distinguished by the plot symbols. Data from the different adapting image sets are shown with different colored symbols: solid yellow for bananas, solid red for carrots, solid green for leaves and open green for cucumbers. The large + symbols, connected to the origin with thick black lines, indicate the mean over all observers and the standard error of that mean. Their color again corresponds to the image set (with a dashed cross for cucumbers). The thin colored dashed lines indicate the vectors towards the mean chromaticities of the original versions of the images used as adaptors, and are displayed in that color

We are specifically interested in the effect that the adapting images had on the chromatic bias (or achromatic point), in relation to the controls. This is shown by a shift in the position of the peaks of the psychometric functions (difference between the solid and dotted functions in Fig. 4), which will be accompanied by a change in height (since they must pass through Pr(“*p* + *t*^″^) = 0.5 at the chromaticity of the pedestal). Our experimental prediction, in the case of the ‘normalization’ explanation, is that the curves will shift in the direction towards the implied color of the adapting images. For example, this would generally be in the + *b*^∗^ direction for bananas. Indeed, such a shift can be seen in Fig. 4 as a rightward shift from the solid curves to the dashed curves. To obtain a measure of these shifts, we take the fitted *μ* parameters of the fitted curves and calculate the difference between these parameters for data from corresponding adapter and control conditions. We average the three separate shifts obtained from the three pedestals within each combination of other variables. This gives us two-dimensional (*a*^∗^, *b*^∗^) shift estimates for each observer and for each image category. These shifts are plotted in Fig. 5, where the directions and magnitudes can be seen, separately for each observer and as an average over observers. Particularly for the bananas and carrots image sets, the achromatic shift is in a direction in color space close to the direction of the predominant color of the original images. This is true for all the individual observers, with some variation. One observer shows a very small shift in the carrots condition, and this reduces the size of the mean shift vector for that condition. For the leaves and cucumbers adapting image sets, the magnitude of the shift is often not as great as for the bananas and carrots, and the direction is not consistent with that of the typical green colors of leaves and cucumbers.

The fitted curves also allow us to estimate the just-noticeable-differences (JNDs) from the *σ* parameter. These were fairly consistent within each observer, and the average over all conditions and observers was 0.74.

## Discussion

We have shown that biases in color perception occur after viewing achromatic images of objects that typically have a specific color. In some cases, this bias is similar in effect to the adaptation that occurs after adaptation to chromatic stimuli. The perceptual shift was measured against a control condition in which the only difference was in the Fourier phase components of the adapting images. Since we compare chromatic judgements after viewing the images of objects to judgements after viewing the same images with phase scrambling, we control for any differential adaptation that might arise from the differences in the spatial contrast sensitivity of the chromatic mechanisms in the peripheral visual system (Mullen, 1985). Since the phase information itself is unlikely to have a biasing effect on color perception, we must assume that the biases we observe come from the semantic content in the non-scrambled images.

### How the visual system might use implied color

For at least some of the stimulus image sets (bananas and carrots), the bias is consistent with the memory color of objects having a normalizing input to chromatic perception, by adapting mechanisms that respond to implied chromatic input. The chromatic direction in which the neutral point shifts is, on average, towards the typical colors of the objects in the adapting images. There is some spread around these directions, but this is to be expected since the observers never saw the original colors of the images and there are likely to be differences between individuals. However, the magnitudes of the shift for the carrots images are smaller than that of the bananas. Furthermore from the two image sets of typically green objects, leaves and cucumbers, we again see generally smaller biases than those from bananas and these biases are in various directions. Many show a shift in the roughly red-purple direction. Considering only the directions of the shifts, this is opposite to what would be predicted by normalization to the implied green, and more consistent with a color constancy illuminant compensation paradigm in which the visual system is discounting a red illuminant (responsible for making the green objects achromatic). It is not immediately clear why the different adapting images have effects consistent with different explanations, however there are some points to highlight.

That the visual system explicitly estimates the illuminant and discounts it is questionable (for evidence against, see Granzier, Brenner, & Smeets, 2009; Rutherford & Brainard 2002), and by no means the only way that color constancy might be achieved. Even if such a mechanism were in operation, we would expect that any estimation of a biased illumination from our adaptation stimuli would be quite weak. It is highly unlikely for a color-biased scene, such as any of the original images we used before they were transformed to greyscale, to be rendered completely achromatic and varying only in luminance by any plausible illumination. Such an illumination would need to be accurately specified both spectrally and spatially to achieve this. Additionally, as mentioned above, the addition of diagnostic color objects to scenes has been shown to improve color constancy very little. Perhaps more importantly, estimating the illuminant in this way would be weakened by other putative mechanisms of color constancy that operate on the lower-level chromatic information in the stimuli. One of the likely ways that the illuminant is estimated is from the average chromaticity of the scene, assuming that on average surfaces have neutral reflectance spectra (e.g., Land 1983, 1986). This would result in a neutral estimate from all our adaptation stimuli, including the controls, and so would not produce any shift in the neutral point. Our scenes do not include objects other than the ones we selected, so do not show the chromatic bias that would be introduced by a biased illuminant.

The typical yellow of bananas lies close to the yellow region of the daylight axis, which runs in a roughly blue to yellow direction (parallel to the *b*^∗^ axis in Fig. 5). It has been suggested that color constancy uses natural illuminants as a prior, and more readily compensates for illuminants that vary in chromaticity on the daylight axis (Brainard, Longere, Delahunt, Freeman, Kraft, & Xiao, 2006; Pearce, Crichton, Mackiewicz, Finlayson, & Hurlbert, 2014), but this is still under debate (e.g., Delahunt & Brainard 2004). Witzel et al., (2011) found memory color effects that were stronger for colors more closely aligned with the daylight axis, but Olkkonen et al., (2008) reject this explanation for their results. In our case, the direction of bias after adapting to images of bananas is inconsistent with a mechanism in which the visual system is discounting an illuminant. Illuminant-compensation might be responsible for some of the results from the typically green adaptors but the direction of shift, while compatible with the colors involved, is roughly orthogonal to the daylight axis and so does not benefit from any increased compensation possible in this direction.

One reason why testing the natural illuminants hypothesis is difficult in this context is that we know of no studies that use images of natural objects that are typically blue, or at least blue but still identifiable when made grayscale. Perhaps the most obvious example of blue in nature is the sky, but an image containing only blue sky would likely not be identifiable when converted to greyscale. Memory color for blue objects has been investigated with man-made objects (Bannert and Bartels, 2013; Bloj et al., 2016; Witzel et al., 2011), but this would have been difficult to achieve in our experiment in which we chose to use natural objects so that they would be familiar to any observer, and many images filled with many examples of those objects.

It is possible that both a normalization mechanism and an illuminant discounting mechanism are in operation and working against each other. If this is the case, then they are unlikely to be perfectly balanced in their effects and the perceptual biases we see are the net effects. In the cases of the bananas and carrots, the implied-color adaptation has a greater effect than the illuminant compensation, and vice-versa for the leaves images. It is not clear why this should be the case, but we do note below that bananas seem to produce particularly strong memory-color effects. It is also conceivable that the two mechanisms operate in sequence, for example with illuminant estimation from inputs taking place after the effects of adaptation to implied color, and again interacting based on the relative effectiveness of each. However, we do not believe that we can make any interpretations about the sequence of operation from this experiment or dataset. They must depend on signals originating after the stage at which objects are recognized, however.

We describe the shift directions from our green-implying adaptors as being opposite to that expected from adaptation, but this is only the case if we consider a traditional color space with opponent axes, as a representation analogous to the peripheral color-vision mechanisms. If we are accomplishing something like adaptation, we have no reason to assume that this occurs at such a peripheral opponent stage. Conceivably, the adaptation might occur at a higher-order (i.e., more central) stage, which does not have the same opponent properties. As mentioned above, systems composed of many narrowly tuned mechanisms can have adapting effects in the opposite direction to normalisation and the suggested central site might encode hue in this way. However, it is still difficult to imagine how adaptation to a “green” signal would result in the achromatic point moving in the red direction, yet a “yellow” signal moves it in the yellow direction. It is possible that the variation in strength and consistency that we see in our measured adaptation effects is because the implied color of some stimuli, bananas in particular, closely matches the preferred color of a particular higher-order mechanism in all our observers, more so than cucumbers. It is also quite possible that using chromatic directions aligned with the adapted mechanisms, rather than the axes of CIE L*a*b* that we arbitrarily chose to test, would reveal more consistent effects, but since we know little about this hypothetical stage of the color visual system this would be challenging.

Any perceptual effect from implied color must come from an observer’s knowledge of the typical colors of objects, gained through experience. The colors of natural objects such as the ones in our stimuli vary, but we expect the experience with them to be relatively consistent among our observers. All observers grew up and live in the United Kingdom, where bananas are almost always yellow when seen for sale. Similarly, carrots are almost always orange. Cucumbers are common, and similarly shaped items (e.g., courgette, marrow) are also green. Leaves, on the other hand, are often various stages of red, orange, or brown. The majority of data were collected in the spring and summer months when the vast majority of leaves in the United Kingdom are green, but nevertheless this may be a source of the inconsistency in the adapting direction and magnitude of the leaves. Furthermore, leaves are often the background elements of a scene, rather than the useful object to be interacted with. This is particularly true when we consider the evolutionarily relevant task of searching for fruit amongst foliage. It is possible that the leaves have little adaptive effect because they are not judged as relevant, and therefore attended to. Perhaps the memory-color effects from the green objects we chose are simply much weaker than those of the bananas and carrots, and this is why we see smaller and much more variable shifts.

### Is the effect real?

The sizes of all the shifts are very small in relation to the often-given size of one JND in CIE *L*^∗^*a*^∗^*b*^∗^ space, which is 1.0 (although could be considerably larger, Mahy, Van Eycken, & Oosterlink, 1994). However, compared to the JNDs specific to our task estimated from the data, the shifts do not seem as small. For example: the magnitude of the shift was 69% of the corresponding JND, on average, for the *b*^∗^ shift with the bananas images. We did not expect to find large effects, since viewing achromatic images does not lead to obviously visible after-effects that are noticeable in normal viewing, as is also true for implied-motion stimuli. However, despite considerable variation between observers, the chromatic biases we measured do seem to be in generally consistent directions for all observers for the bananas and carrots images sets.

The conflicting directions of the adapting effects, in some cases, serves to weaken the evidence for adapting effects from implied color. However, we do not believe that this is a problem with our methodology. The ‘roving pedestal’ 2AFC method (Morgan et al., 2013) that we use is ideally suited to an experiment of this kind since it allows us to be much more certain that our measurements are those of perceptual biases. Had the observer been asked to simply make a judgement of a single stimulus, then they might have based their response on their expectation after identifying the objects in the adapting set, which could be a symbolic cue. Had the pedestal chromaticity always been the same, they may have been able to identify it on each trial and this may have influenced the decision. In addition, an adjustment task in which the observer was required to adjust a stimulus so that it appeared neutral or the same as their memory color of an object would have not been appropriate, since viewing that stimulus itself for any period of time would likely lead to low-level adaptation effects greater than the effects that we sought.

### Comparison to other studies

We know of no other work that has attempted to show adaptation to implied color or memory color, or that the effect of the color implication lasts beyond the presentation of the achromatic implying stimulus. We measure changes in perception not of the memory color objects themselves, but on simple geometric stimuli that are not presented at the same time. We also use a different task that avoids lengthy response periods, avoids response bias, and does not explicitly measure an achromatic percept. However, there are some other experiments that show similar features to ours in their results. In particular, Olkkonen et al., (2008) found smaller amounts of “over compensation” when observers adjusted the color of a variety of images of fruit and vegetables, including carrots and those that are typically green, to appear achromatic than when they adjusted the color of bananas. We also see a similarly weak effect from our other images, relative to bananas. Indeed, bananas are commonly used as stimuli in memory color experiments (e.g., Tanaka and Presnell 1999; Biederman & Ju 1988; Bannert & Bartels 2013; Olkkonen et al., 2008; Granzier & Gegenfurtner 2012; Yendrikhovskij, Blommaert, & de Ridder, 1999; Vurro, Ling, & Hurlbert, 2013), sometimes they are the only real object used (Kanematsu & Brainard, 2014; Witzel, 2016), but they are notably absent from (Bartleson, 1960). If we had limited our stimulus images to bananas, we might have drawn stronger conclusions. It is unclear as to why bananas illicit a particularly strong memory color, since they change color (from green to yellow to brown) as they ripen just as many other fruit. It might be suggested that since the typical yellow of bananas lies close to the daylight axis, an illuminant bias is more readily accepted as a reason for a change in their chromaticity but, as stated above, this seems inconsistent with the results of our experiment.

### Source of the top-down signal

Our experiment took the form of a typical visual adaptation experiment, and we present observers with greyscale images of objects with a typical color in the assumption that these images will trigger the sensation of that color. However, it might be argued that these images are not necessary, and we need only ask our observers to imagine an object without ever seeing it. Observers are able to reproduce or select a color according to their internal representation from a verbal instruction (Bartleson 1960; Pérez-Carpinell, De Fez, Baldoví, & Soriano, 1998), and perhaps a command to imagine an object, or even simply a color, would be sufficient to cause an adapting effect. This experiment remains to be performed, but since the stimuli described do not imply an illuminant, it might provide a stronger means to determine between a normalisation vs. illuminant compensation source of the effect. However, when other researchers used stimuli that were degraded in their similarity to the real objects (by using silhouettes) they measured weaker effects (Olkkonen et al., 2008), so perhaps an accurate representation of the object is necessary to invoke memory color.

### Conclusions

We provide some evidence that images that do not contain chromatic information, but which imply color through the memory color of the content of the image, can produce perceptual biases that are similar to chromatic adaptation to the real color. This evidence is weak, however, and we remain cautious in drawing conclusions, particularly when different mechanisms may be working in opposition.

## Electronic supplementary material

Below is the link to the electronic supplementary material.

(PDF 241 KB)

(CSV 929 KB)
