# Supplementary material for: Chromatic adaptation from achromatic stimuli with implied color
Source: Atten Percept Psychophys. 2019 Jun 14;81(8):2890–901. doi: 10.3758/s13414-019-01716-5 (PMC6856295; doi:10.3758/s13414-019-01716-5)
Supplement: Supplementary file 1 — (PDF 241 KB) [file 13414_2019_1716_MOESM1_ESM.pdf]

Supplementary material

Response proportions and fitted psychometric functions from all observers, chromatic directions and stimulus image sets. Each plot's title indicates the image set and observer. Each row of plots shows data from one chromatic direction tested, and the colours of the plot lines and symbols indicate that direction: blue, black and yellow for b*, green, black and red for a*.
On each row, separate panels show data from trials with different pedestal chromaticities: left panel: green or blue ($a^* = -1$ or $b^* = 1$). Centre panel: neutral ($a^* = 0$ or $b * = 0$). Right panel: yellow or red ($a^* = 1$ or $b * = 1$).
The vertical axes are the proportions of times that the observer responded that the pedestal plus test stimulus (as opposed to the pedestal) was more neutral, for each of the pedestal plus test chromaticities on the horizontal axis. Data for the control condition (phase-scrambled images) are shown with square symbols and fitted with solid curves. Data from the image condition are shown with x symbols and fitted with dashed curves. Error bars show 95% confidence intervals. Vertical dotted lines indicate the chromaticities of the pedestals, and the horizontal dotted line indicates the proportion at which the observer is equally likely to choose the pedestal plus test or pedestal.
The r^2 values on each plot indicate the goodness-of-fit of each of the curves. The upper r^2 value corresponds to the control condition (dashed curve) and the lower r^2 value corresponds to the image condition (solid curve).

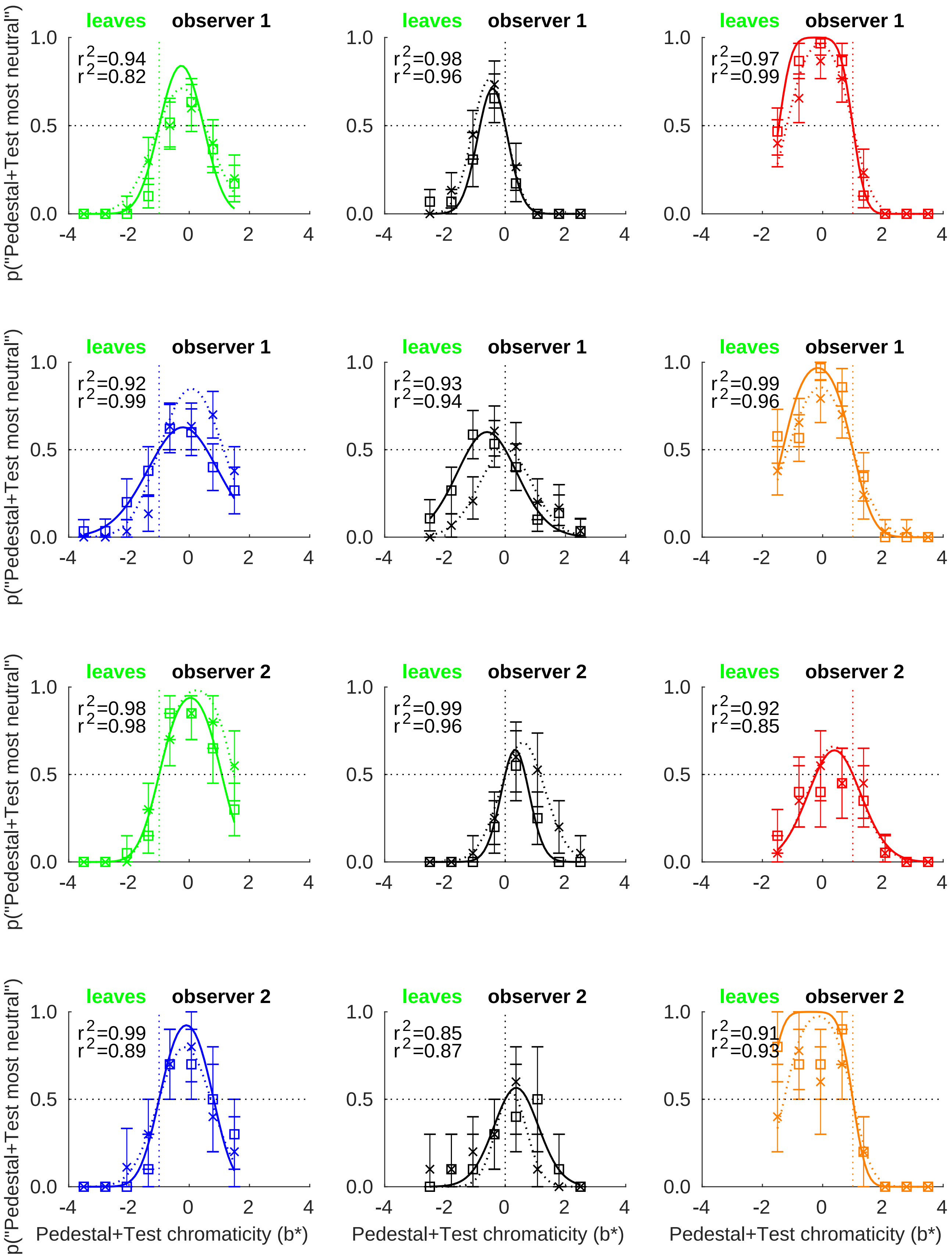
leaves observer 1
r2=0.94
r2=0.82
r2=0.98
r2=0.96
r2=0.97
r2=0.99
r2=0.92
r2=0.99
r2=0.93
r2=0.94
r2=0.99
r2=0.96
leaves observer 2
r2=0.98
r2=0.98
r2=0.99
r2=0.96
r2=0.92
r2=0.85
r2=0.99
r2=0.89
r2=0.85
r2=0.87
r2=0.91
r2=0.93
p("Pedestal+Test most neutral")
Pedestal+Test chromaticity (b*)
1.0
0.5
0.0
-4
-2
0
2
4

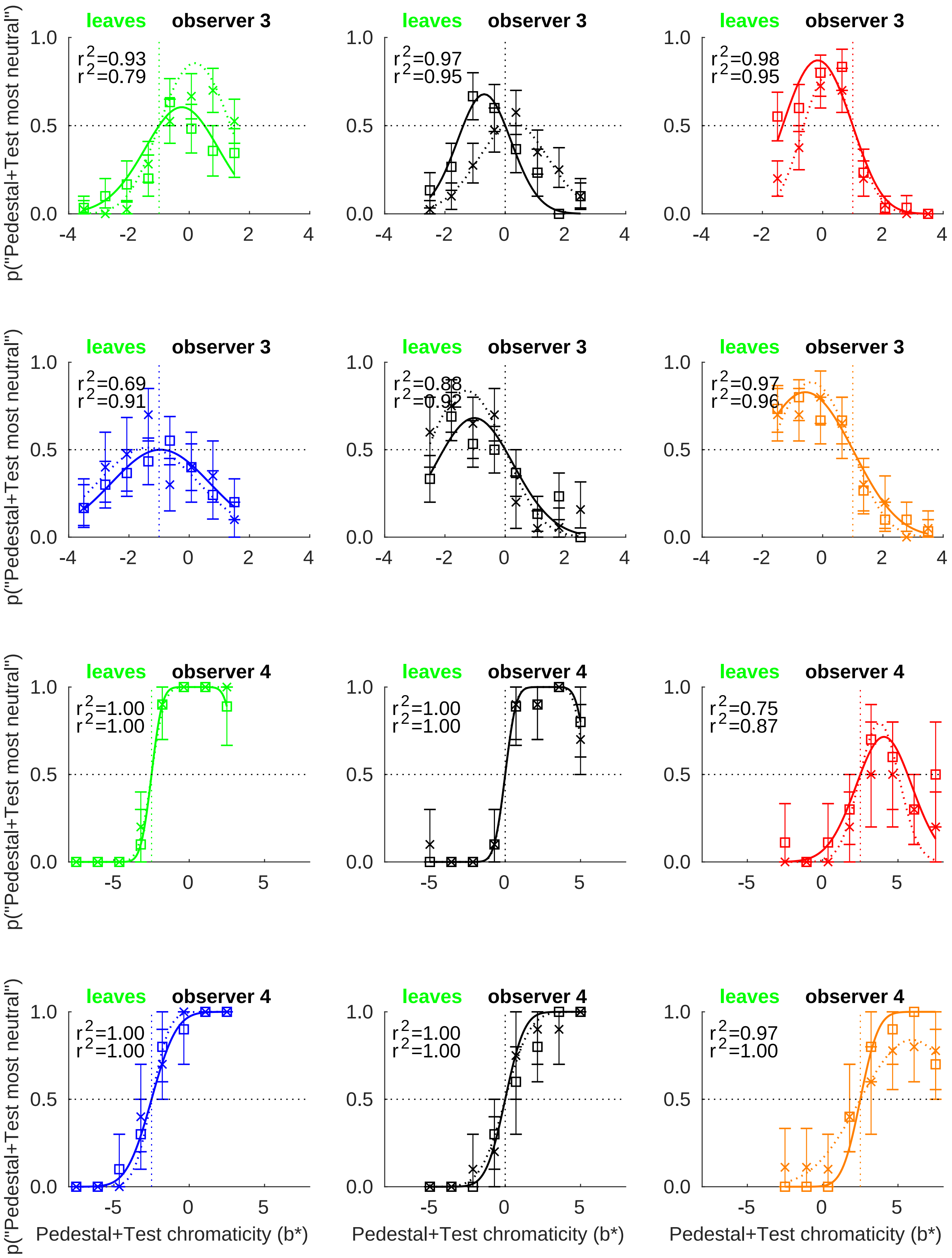

leaves
observer 3
r2=0.93
r2=0.79
leaves
observer 3
r2=0.97
r2=0.95
leaves
observer 3
r2=0.98
r2=0.95
leaves
observer 3
r2=0.69
r2=0.91
leaves
observer 3
r2=0.88
r2=0.92
leaves
observer 3
r2=0.97
r2=0.96
leaves
observer 4
r2=1.00
r2=1.00
leaves
observer 4
r2=1.00
r2=1.00
leaves
observer 4
r2=0.75
r2=0.87
leaves
observer 4
r2=1.00
r2=1.00
leaves
observer 4
r2=1.00
r2=1.00
leaves
observer 4
r2=0.97
r2=1.00
p("Pedestal+Test most neutral")
Pedestal+Test chromaticity (b*)

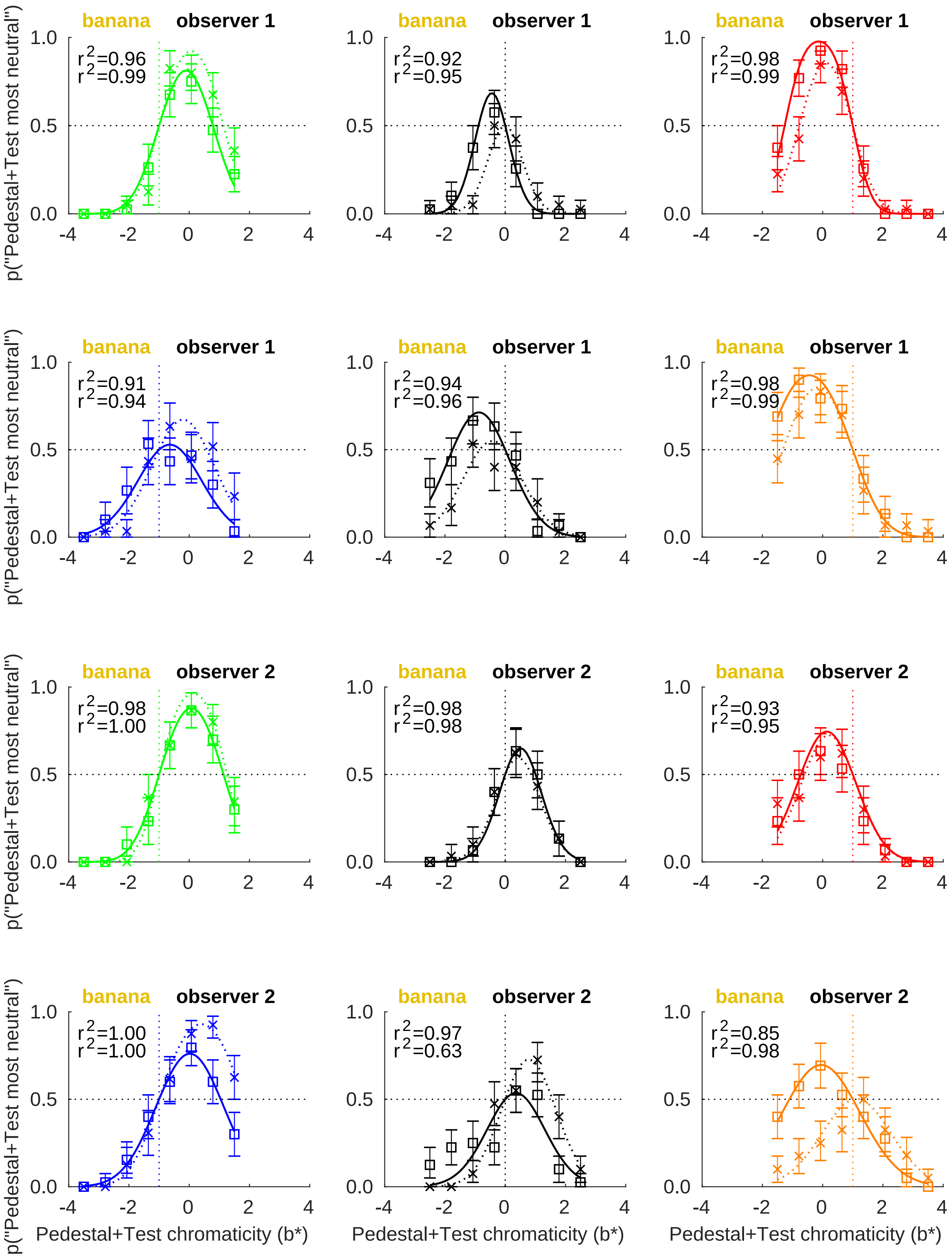
banana
observer 1
r2=0.96
r2=0.99
r2=0.92
r2=0.95
r2=0.98
r2=0.99
r2=0.91
r2=0.94
r2=0.94
r2=0.96
r2=0.98
r2=0.99
observer 2
r2=0.98
r2=1.00
r2=0.98
r2=0.98
r2=0.93
r2=0.95
r2=1.00
r2=1.00
r2=0.97
r2=0.63
r2=0.85
r2=0.98
p("Pedestal+Test most neutral")
Pedestal+Test chromaticity (b*)

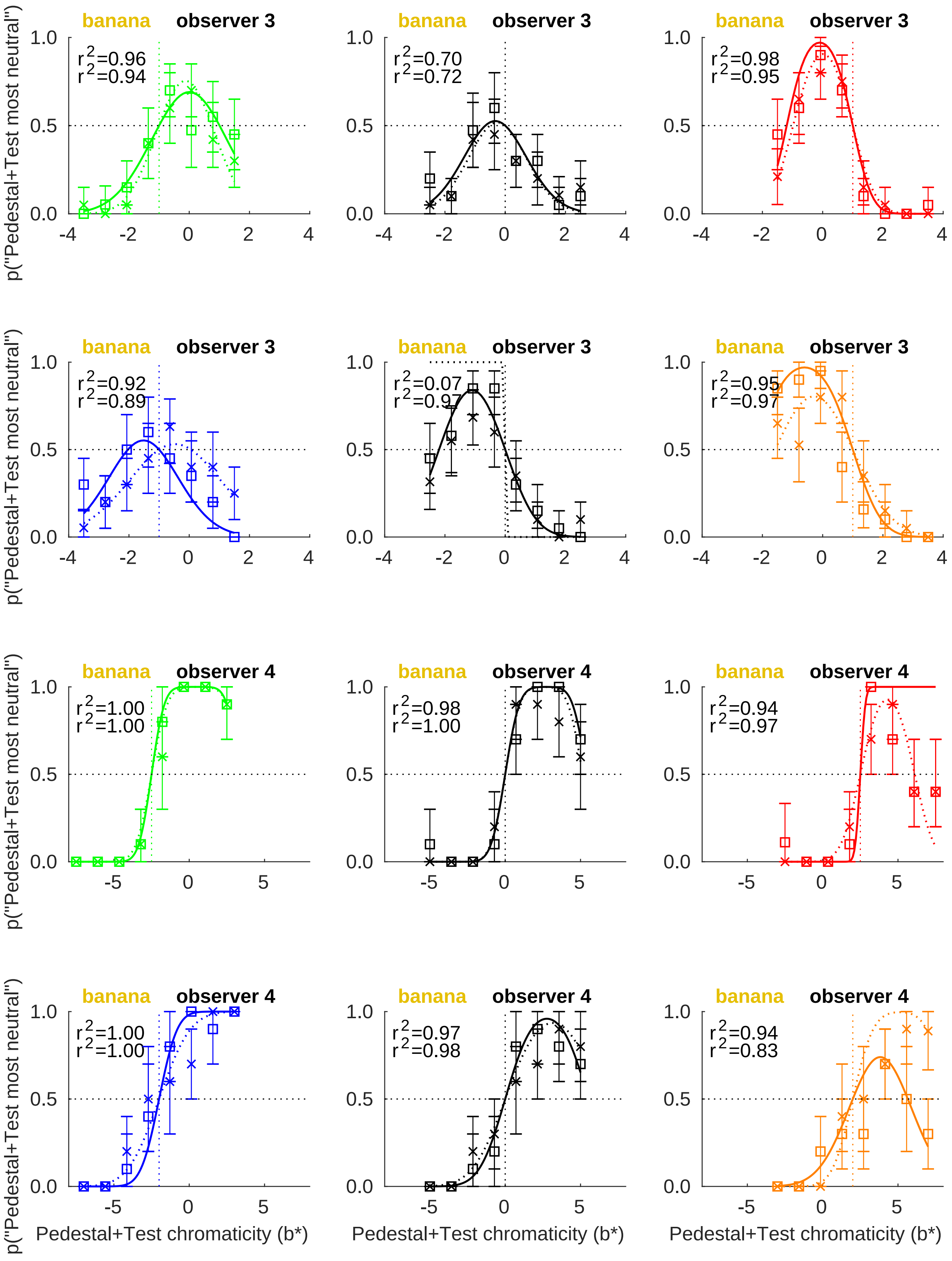
banana
observer 3
r2=0.96
r2=0.94
r2=0.70
r2=0.72
r2=0.98
r2=0.95
r2=0.92
r2=0.89
r2=0.07
r2=0.97
r2=0.95
r2=0.97
observer 4
r2=1.00
r2=1.00
r2=0.98
r2=1.00
r2=0.94
r2=0.97
r2=1.00
r2=1.00
r2=0.97
r2=0.98
r2=0.94
r2=0.83
p("Pedestal+Test most neutral")
Pedestal+Test chromaticity (b*)

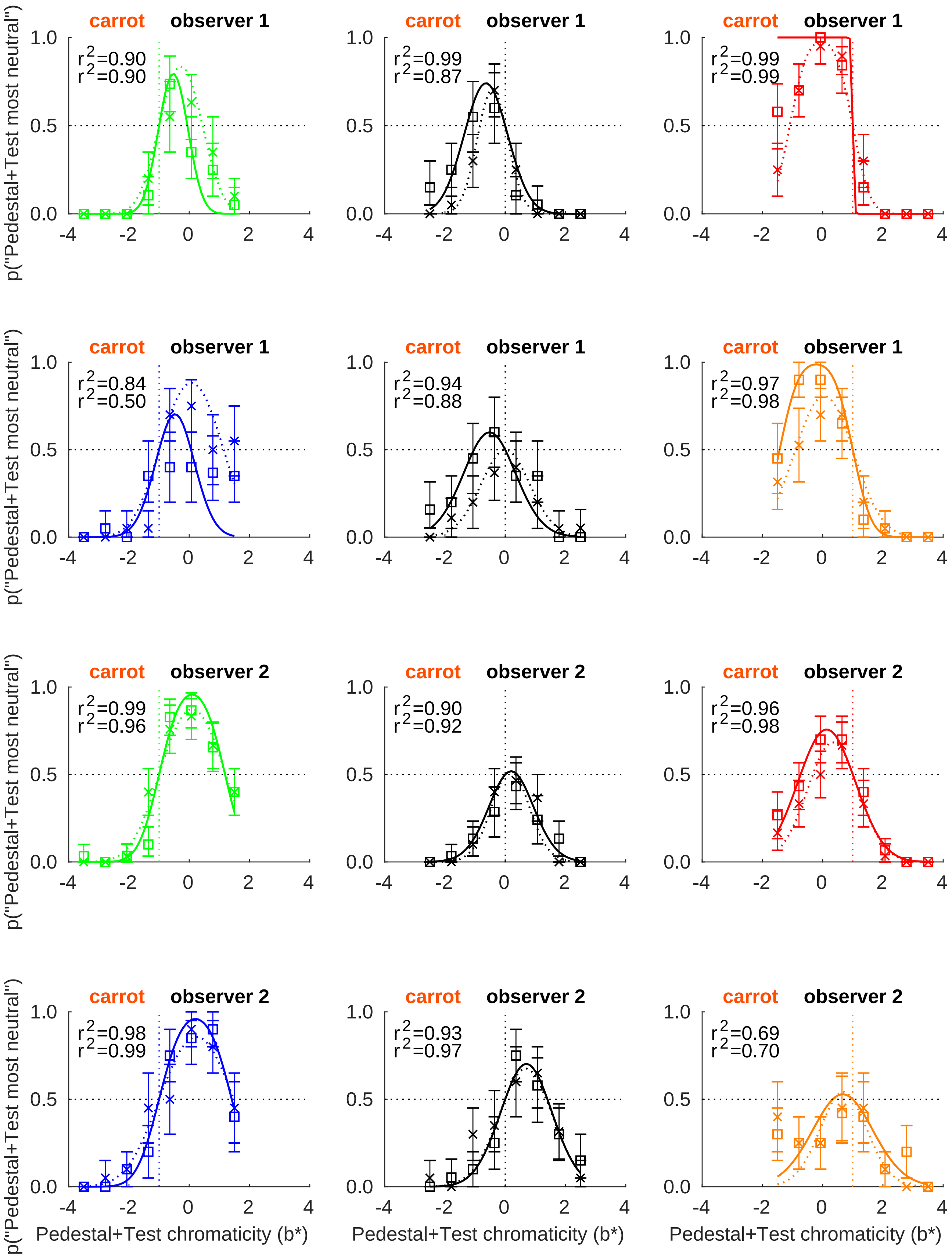
carrot observer 1
r2=0.90
r2=0.90
carrot observer 1
r2=0.99
r2=0.87
carrot observer 1
r2=0.99
r2=0.99
carrot observer 1
r2=0.84
r2=0.50
carrot observer 1
r2=0.94
r2=0.88
carrot observer 1
r2=0.97
r2=0.98
carrot observer 2
r2=0.99
r2=0.96
carrot observer 2
r2=0.90
r2=0.92
carrot observer 2
r2=0.96
r2=0.98
carrot observer 2
r2=0.98
r2=0.99
carrot observer 2
r2=0.93
r2=0.97
carrot observer 2
r2=0.69
r2=0.70
p("Pedestal+Test most neutral")
Pedestal+Test chromaticity (b*)
0.0
0.5
1.0
-4
-2
0
2
4

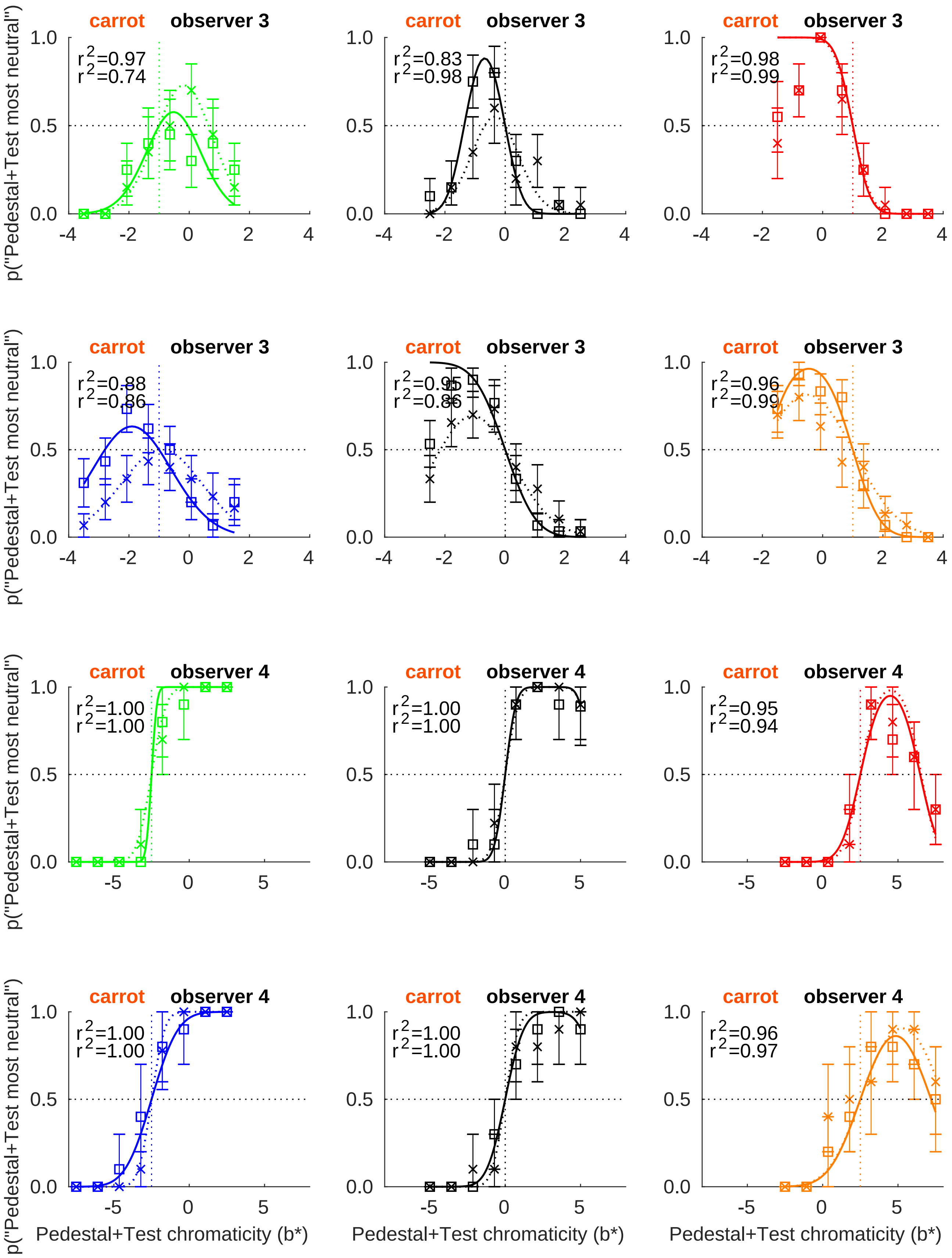
carrot observer 3
r2=0.97
r2=0.74
carrot observer 3
r2=0.83
r2=0.98
carrot observer 3
r2=0.98
r2=0.99
carrot observer 3
r2=0.88
r2=0.86
carrot observer 3
r2=0.95
r2=0.86
carrot observer 3
r2=0.96
r2=0.99
carrot observer 4
r2=1.00
r2=1.00
carrot observer 4
r2=1.00
r2=1.00
carrot observer 4
r2=0.95
r2=0.94
carrot observer 4
r2=1.00
r2=1.00
carrot observer 4
r2=1.00
r2=1.00
carrot observer 4
r2=0.96
r2=0.97
p("Pedestal+Test most neutral")
Pedestal+Test chromaticity (b*)
1.0
0.5
0.0
-4
-2
0
2
4
-5
5

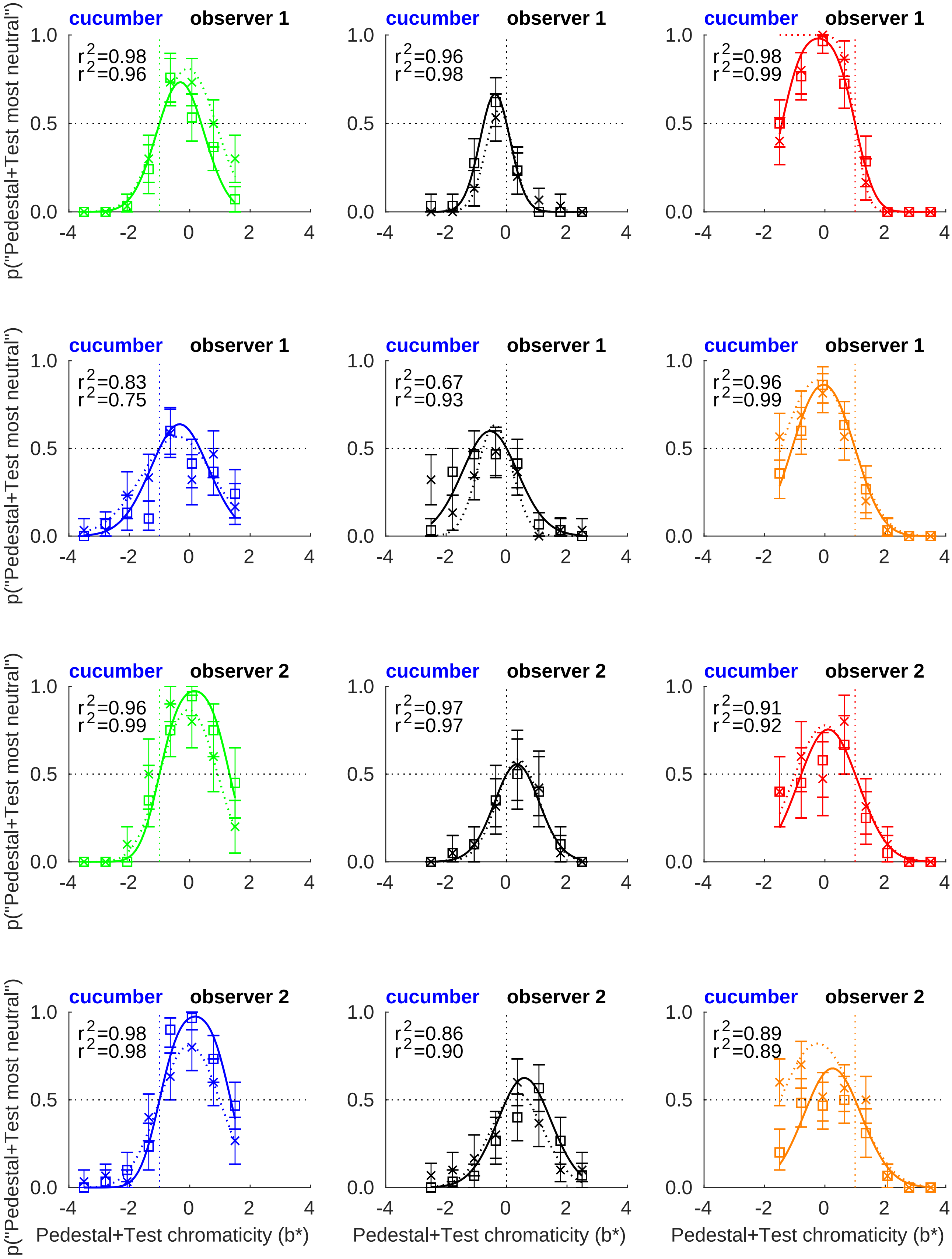

cucumber
observer 1
r2=0.98
r2=0.96
r2=0.96
r2=0.98
r2=0.98
r2=0.99
r2=0.83
r2=0.75
r2=0.67
r2=0.93
r2=0.96
r2=0.99
observer 2
r2=0.96
r2=0.99
r2=0.97
r2=0.97
r2=0.91
r2=0.92
r2=0.98
r2=0.98
r2=0.86
r2=0.90
r2=0.89
r2=0.89
p("Pedestal+Test most neutral")
Pedestal+Test chromaticity (b*)

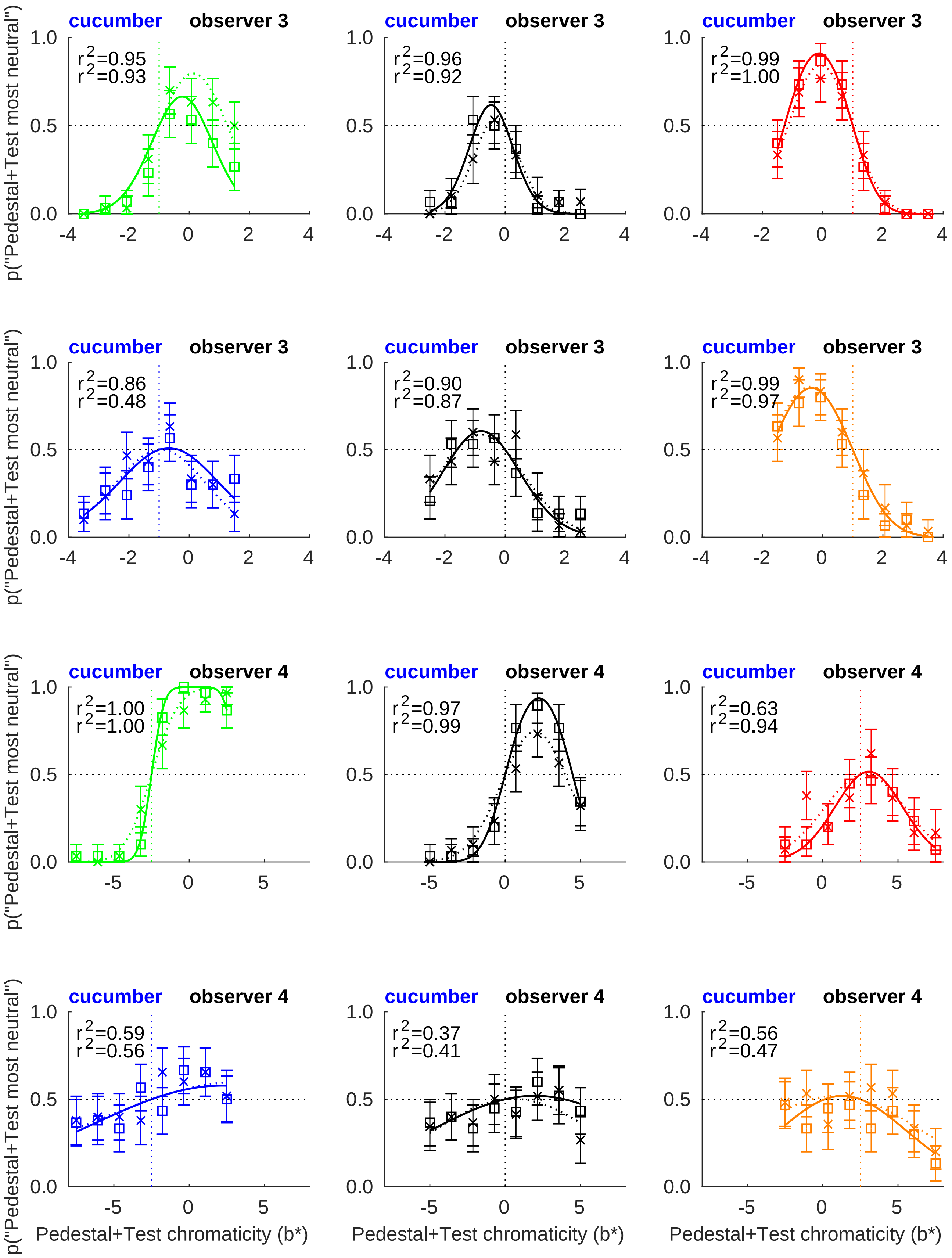
cucumber observer 3
r2=0.95
r2=0.93
r2=0.96
r2=0.92
r2=0.99
r2=1.00
r2=0.86
r2=0.48
r2=0.90
r2=0.87
r2=0.99
r2=0.97
cucumber observer 4
r2=1.00
r2=1.00
r2=0.97
r2=0.99
r2=0.63
r2=0.94
r2=0.59
r2=0.56
r2=0.37
r2=0.41
r2=0.56
r2=0.47
p("Pedestal+Test most neutral")
Pedestal+Test chromaticity (b*)
1.0
0.5
0.0
-4
-2
0
2
4
-5
5
